# Perianal Giant Condyloma Acuminatum—Buschke-Löwenstein Tumor: A Case Report

**DOI:** 10.1155/2012/507374

**Published:** 2012-11-20

**Authors:** Sukru Tas, Muhammet Kasim Arik, Faruk Ozkul, Oztekin Cikman, Yilmaz Akgun

**Affiliations:** Department of General Surgery, Faculty of Medicine, Çanakkale Onsekiz Mart University, Floor 2, 17100 Çanakkale, Turkey

## Abstract

Condyloma acuminatum caused by Human Papillomavirus is the most commonly occurring sexually transmitted infection in the anogenital region. Buschke-Löwenstein tumor (BLT) known also as giant condyloma acuminatum is a rare disease. The disease, for which the most important treatment method is the surgical excision, differs from normal condyloma acuminatum cases with its high degree of malignancy. The purpose of this paper is to present the case that reached huge dimensions in the perianal region and that was treated with wide resection in the literature.

## 1. Introduction

Condyloma acuminatum is a disease in which Human Papillomavirus (HPV) is active, that shows epithelial overgrowth. It is most commonly seen in genital, anal, and perianal regions. It is the most common sexually transmitted infection of anorectal region [1]. Incidence rate in general population is 0.1% [2]. 2-3 million new cases are added every year to condyloma acuminatum especially with growing numbers in homosexual male population [3].

Incubation period of HPV that causes condyloma acuminatum is 1–6 months. Giant condyloma acuminatum known also as Buschke-Löwenstein tumor (BLT), is a slow growing, although histopathologically benign, clinically malignant rare disease. The most important treatment method is the excision of the mass. To determine the margins of the mass, degree of invasion, lymph node involvement, and whether the case is primary or recurrence before the surgery shall identify the method of surgical treatment.

The purpose of this paper is to present a BLT case that reached huge dimensions covering all perianal region and extending into anal channel and developed due to condyloma acuminatum.

## 2. Case Presentation

**Figure 1:**
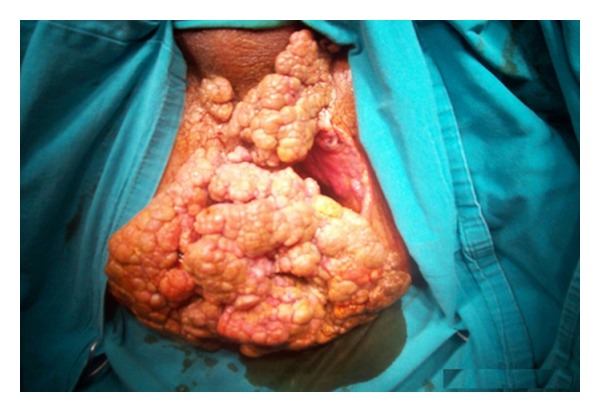
BLT covering all the perianal region.

**Figure 2:**
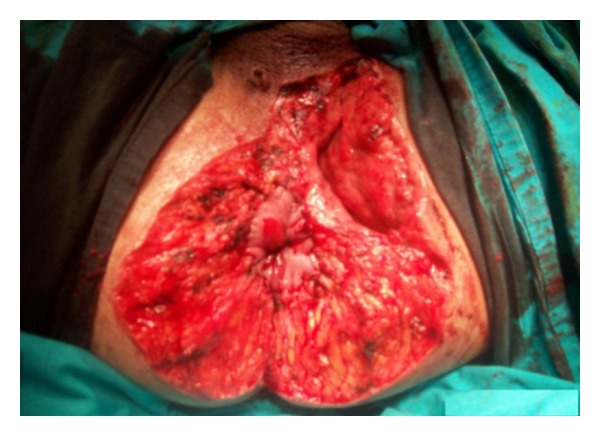
Image of the perianal region after the excision of the mass.

**Figure 3:**
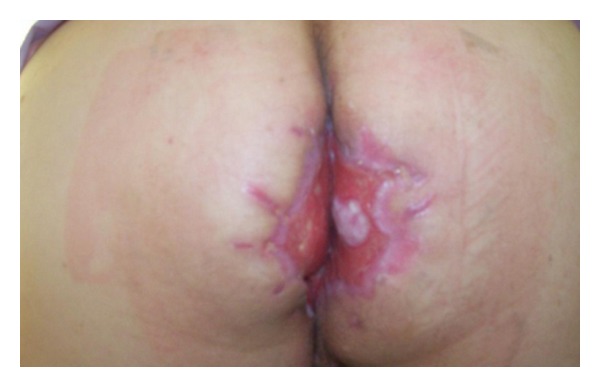
The image after healing of the case.

53-year-old-male patient was applied with the complaint of palpable mass that originated 10 years ago and gradually enlarged, causing discharge with unpleasant odor, recently causing gas-fecal incontinence. His history did not reveal any characteristics. In the examination of the patient, 20 × 20 cm mass was observed which was covered with exudate with unpleasant odor, shaped like broccoli, and grew towards scrotum and penis root covering all perianal region beginning from anal channel (Figure 1). Colonoscopic examination showed that the mass reached linea dentata and enclosed mucosa. Magnetic resonance imaging of the patient showed that the mass did not invade anal sphincter and was localized to anal mucosa. Laboratory findings did not reveal any feature other than high leucocyte count (WBC: 14000). Local excision of the mass with negative surgical margins in lithotomy position and general anesthesia was applied to the patient. Findings of peroperative infection were determined and excision region was left open for secondary healing (Figure 2). Then operation was terminated after the laparoscopic loop colostomy was performed. After histopathological examination of the mass, it was reported as condyloma acuminatum and invasive tumor was not observed in the mass. Chemoradiotherapy was not planned because malignant transformation was not detected in the patient. Colostomy of the patient was closed 6 months later after the excision region is secondarily healed (Figure 3) and its sphincter tone was evaluated as normal in manometric studies. There was not any complaint or recurrence in the patient during postoperative 6-month follow-up period.

## 3. Discussion

Condyloma acuminatum is a benign disease caused by Human Papillomavirus that is sexually transmitted and that can cause malignant transformation. In HPV transmission homosexuality, bad genital hygiene, chronic genital infections, and polygamy can be considered as risk factors. BLT is a rarely seen form which develops by the overgrowth of condyloma acuminatum and has a high risk of malignant transformation [4]. BLT is considered pathologically to be in between condyloma acuminatum and perianal squamous cell carcinoma. Although squamous cell carcinoma in perianal region is clinically similar to condyloma acuminatum and giant condyloma acuminatum, in histopathological examination squamous cell carcinoma differs from the other two with regard to malignant proliferation. However, BLT shows similarity to malignity with deep invasion to the tissue below, fast mitotic activity, and proliferation. Most commonly seen clinical symptoms are pelvic pain, perianal secretion, anorectal bleeding, and the impairment of anal sphincter continence. BLT's local recurrence rate is high. Malignant transformation occurs in approximately 30–50% of the patients. Chronic alcoholism, immune suppressing medicine, and diseases increase the recurrence and malignant transformation risk.

Most efficient treatment method especially during the early period of disease is a surgical excision [5]. Systemic or topical chemotherapy and radiotherapy can be applied to patients to whom surgical operation cannot be performed. This method is mostly considered in irresectable cases for palliative treatment after incomplete excision and in recurrent cases [6]. In cases where rectum and anal sphincter muscles are invaded, recurrence or malignant transformation is developed, there are various treatment options as abdominoperineal resection. Most of the authorities suggest a temporary loop colostomy to be opened to prevent fecal contamination in the wound before wide surgical excision [7]. Since our case presented only anal mucosa involvement and malign transformation was not detected, wide local excision was chosen. Laparoscopic loop sigmoidostomy was performed that is frequently used nowadays to prevent fecal contamination. Since malign transformation was not observed in the histopathology of excised piece, chemoradiotherapy or any other new surgical invasion was not planned for the patient after surgery.

## 4. Conclusion

Early surgical resection in the treatment of condyloma acuminatum prevents the development of BLT. It is necessary to determine histopathologically whether a malignant transformation occurred, or whether the anal sphincter muscles and rectum are invaded with radiological and endoscopic imaging in BLT cases before the surgery for determining the surgical method.

## References

[1] Balik E, Eren T, Bugra D (2009). A surgical approach to anogenital Buschke Loewenstein tumours (giant condyloma acuminata). *Acta Chirurgica Belgica*.

[2] Paraskevas KI, Kyriakos E, Poulios EE, Stathopoulos V, Tzovaras AA, Briana DD (2007). Surgical management of giant condyloma acuminatum (Buschke-Löewenstein tumor) of the perianal region. *Dermatologic Surgery*.

[3] Renzi A, Giordano P, Renzi G, Landolfi V, del Genio A, Weiss EG (2006). Buschke-Löwenstein tumor successful treatment by surgical excision alone: a case report. *Surgical Innovation*.

[4] Chu QD, Vezeridis MP, Libbey NP, Wanebo HJ (1994). Giant condyloma acuminatum (Buschke-Löwenstein tumor) of the anorectal and perianal regions: analysis of 42 cases. *Diseases of the Colon and Rectum*.

[5] Paipiu HS, Duminici A, Olariu T (2011). Perianal giant condyloma acuminatum (Buschke-Löwenstein tumor). Case report and review of literature. *Chirurgia*.

[6] Wietfeldt ED, Thiele J (2009). Malignancies of the anal margin and perianal skin. *Clinics in Colon and Rectal Surgery*.

[7] de Toma G, Cavallaro G, Bitonti A, Polistena A, Onesti MG, Scuderi N (2006). Surgical management of perianal giant condyloma acuminatum (Buschke-Löwenstein tumor): report of three cases. *European Surgical Research*.

